# Supplementary material for: Modeling glioblastoma heterogeneity as a dynamic network of cell states
Source: Mol Syst Biol. 2021 Sep 16;17(9):e10105. doi: 10.15252/msb.202010105 (PMC8444284; doi:10.15252/msb.202010105)
Supplement: Supplementary file 6 — Source Data for Figure 5 [file MSB-17-e10105-s004.zip › Figure5A_sourcedata/GSEA_3017/nosigdb_stateC.GseaPreranked.1622795002785/RICHARDS_NOWAKOWSKI_SCIENCE_2017_ENDOTHELIAL_UPREG.html]

Details for gene set RICHARDS\_NOWAKOWSKI\_SCIENCE\_2017\_ENDOTHELIAL\_UPREG[GSEA]

|  || Dataset | state13017 |
| Phenotype | NoPhenotypeAvailable |
| Upregulated in class | na\_neg |
| GeneSet | RICHARDS\_NOWAKOWSKI\_SCIENCE\_2017\_ENDOTHELIAL\_UPREG |
| Enrichment Score (ES) | -0.4088974 |
| Normalized Enrichment Score (NES) | -3.0130851 |
| Nominal p-value | 0.0 |
| FDR q-value | 0.0 |
| FWER p-Value | 0.0 |
Table: GSEA Results Summary

  

Fig 1: Enrichment plot: RICHARDS\_NOWAKOWSKI\_SCIENCE\_2017\_ENDOTHELIAL\_UPREG      
 Profile of the Running ES Score & Positions of GeneSet Members on the Rank Ordered List

  

| PROBE | GENE SYMBOL | GENE\_TITLE | RANK IN GENE LIST | RANK METRIC SCORE | RUNNING ES | CORE ENRICHMENT || 1 | FAM111A |  |  | 13 | 0.576 | -0.0167 | No |
| 2 | MCM3 |  |  | 114 | 0.324 | -0.2259 | No |
| 3 | FRMD4B |  |  | 123 | 0.313 | -0.2370 | No |
| 4 | SLC12A2 |  |  | 148 | 0.288 | -0.2831 | No |
| 5 | UPP1 |  |  | 204 | -0.253 | -0.3966 | No |
| 6 | CAST |  |  | 205 | -0.253 | -0.3917 | No |
| 7 | STC1 |  |  | 210 | -0.255 | -0.3953 | No |
| 8 | TNFAIP8L1 |  |  | 215 | -0.257 | -0.3989 | No |
| 9 | NPAS2 |  |  | 219 | -0.258 | -0.4003 | No |
| 10 | ARL4A |  |  | 224 | -0.259 | -0.4038 | Yes |
| 11 | IGFBP7 |  |  | 225 | -0.259 | -0.3987 | Yes |
| 12 | TPST2 |  |  | 226 | -0.259 | -0.3937 | Yes |
| 13 | FERMT2 |  |  | 227 | -0.259 | -0.3886 | Yes |
| 14 | ATOX1 |  |  | 229 | -0.260 | -0.3857 | Yes |
| 15 | IGF2BP2 |  |  | 231 | -0.260 | -0.3827 | Yes |
| 16 | MBNL2 |  |  | 233 | -0.260 | -0.3798 | Yes |
| 17 | ZYX |  |  | 236 | -0.261 | -0.3790 | Yes |
| 18 | CLIC1 |  |  | 237 | -0.261 | -0.3739 | Yes |
| 19 | SAT1 |  |  | 241 | -0.262 | -0.3752 | Yes |
| 20 | LIMS1 |  |  | 244 | -0.262 | -0.3744 | Yes |
| 21 | ZFYVE21 |  |  | 247 | -0.263 | -0.3735 | Yes |
| 22 | TPT1 |  |  | 248 | -0.263 | -0.3684 | Yes |
| 23 | AGTRAP |  |  | 257 | -0.266 | -0.3804 | Yes |
| 24 | PPIB |  |  | 265 | -0.268 | -0.3902 | Yes |
| 25 | ANAPC16 |  |  | 268 | -0.269 | -0.3893 | Yes |
| 26 | KIF9 |  |  | 269 | -0.269 | -0.3840 | Yes |
| 27 | RPL41 |  |  | 270 | -0.269 | -0.3787 | Yes |
| 28 | SOCS3 |  |  | 274 | -0.270 | -0.3799 | Yes |
| 29 | EHD2 |  |  | 275 | -0.270 | -0.3746 | Yes |
| 30 | RPS23 |  |  | 282 | -0.271 | -0.3822 | Yes |
| 31 | RRBP1 |  |  | 283 | -0.271 | -0.3769 | Yes |
| 32 | SH3BGRL3 |  |  | 286 | -0.272 | -0.3759 | Yes |
| 33 | UGCG |  |  | 287 | -0.273 | -0.3705 | Yes |
| 34 | BAMBI |  |  | 289 | -0.273 | -0.3673 | Yes |
| 35 | OSMR |  |  | 292 | -0.274 | -0.3663 | Yes |
| 36 | PHLDA1 |  |  | 295 | -0.274 | -0.3652 | Yes |
| 37 | RPS18 |  |  | 301 | -0.277 | -0.3706 | Yes |
| 38 | HERPUD1 |  |  | 302 | -0.277 | -0.3651 | Yes |
| 39 | CNKSR3 |  |  | 305 | -0.280 | -0.3640 | Yes |
| 40 | NAMPT |  |  | 306 | -0.280 | -0.3585 | Yes |
| 41 | LIFR |  |  | 311 | -0.282 | -0.3616 | Yes |
| 42 | NR2F2 |  |  | 313 | -0.283 | -0.3582 | Yes |
| 43 | PDLIM2 |  |  | 314 | -0.283 | -0.3526 | Yes |
| 44 | SERPINE1 |  |  | 323 | -0.286 | -0.3643 | Yes |
| 45 | AHR |  |  | 324 | -0.286 | -0.3587 | Yes |
| 46 | RPL17 |  |  | 329 | -0.287 | -0.3616 | Yes |
| 47 | RPL12 |  |  | 332 | -0.288 | -0.3603 | Yes |
| 48 | EHD4 |  |  | 334 | -0.289 | -0.3568 | Yes |
| 49 | SPATS2L |  |  | 339 | -0.290 | -0.3597 | Yes |
| 50 | PPP1R15A |  |  | 342 | -0.293 | -0.3583 | Yes |
| 51 | RPS2 |  |  | 346 | -0.296 | -0.3590 | Yes |
| 52 | DLC1 |  |  | 347 | -0.296 | -0.3532 | Yes |
| 53 | ABLIM1 |  |  | 348 | -0.297 | -0.3473 | Yes |
| 54 | SHISA5 |  |  | 349 | -0.297 | -0.3415 | Yes |
| 55 | CHST2 |  |  | 350 | -0.297 | -0.3357 | Yes |
| 56 | COL4A2 |  |  | 353 | -0.300 | -0.3341 | Yes |
| 57 | S100A11 |  |  | 361 | -0.301 | -0.3433 | Yes |
| 58 | SPRY1 |  |  | 369 | -0.307 | -0.3524 | Yes |
| 59 | PMEPA1 |  |  | 375 | -0.308 | -0.3571 | Yes |
| 60 | FOS |  |  | 377 | -0.309 | -0.3532 | Yes |
| 61 | CREB3L2 |  |  | 378 | -0.309 | -0.3472 | Yes |
| 62 | APLP2 |  |  | 379 | -0.310 | -0.3411 | Yes |
| 63 | SLC2A1 |  |  | 383 | -0.311 | -0.3415 | Yes |
| 64 | RHOB |  |  | 391 | -0.316 | -0.3504 | Yes |
| 65 | S100A13 |  |  | 396 | -0.319 | -0.3527 | Yes |
| 66 | PLTP |  |  | 397 | -0.319 | -0.3465 | Yes |
| 67 | TIMP1 |  |  | 399 | -0.320 | -0.3424 | Yes |
| 68 | ZFP36L2 |  |  | 401 | -0.321 | -0.3382 | Yes |
| 69 | GADD45A |  |  | 405 | -0.323 | -0.3384 | Yes |
| 70 | DDX21 |  |  | 413 | -0.331 | -0.3470 | Yes |
| 71 | LDLR |  |  | 419 | -0.334 | -0.3512 | Yes |
| 72 | CALD1 |  |  | 420 | -0.335 | -0.3446 | Yes |
| 73 | SLC2A3 |  |  | 422 | -0.336 | -0.3402 | Yes |
| 74 | KLF6 |  |  | 429 | -0.339 | -0.3465 | Yes |
| 75 | INSIG1 |  |  | 434 | -0.342 | -0.3484 | Yes |
| 76 | WWTR1 |  |  | 437 | -0.345 | -0.3460 | Yes |
| 77 | WLS |  |  | 438 | -0.345 | -0.3392 | Yes |
| 78 | HSPA5 |  |  | 447 | -0.350 | -0.3496 | Yes |
| 79 | MYL12A |  |  | 448 | -0.350 | -0.3427 | Yes |
| 80 | RHOC |  |  | 455 | -0.359 | -0.3486 | Yes |
| 81 | EDNRB |  |  | 462 | -0.367 | -0.3543 | Yes |
| 82 | SERPINB6 |  |  | 463 | -0.367 | -0.3471 | Yes |
| 83 | B2M |  |  | 468 | -0.371 | -0.3485 | Yes |
| 84 | PTPRE |  |  | 469 | -0.372 | -0.3412 | Yes |
| 85 | HES1 |  |  | 470 | -0.373 | -0.3339 | Yes |
| 86 | NQO1 |  |  | 479 | -0.381 | -0.3436 | Yes |
| 87 | HBEGF |  |  | 480 | -0.386 | -0.3361 | Yes |
| 88 | RAB13 |  |  | 482 | -0.388 | -0.3306 | Yes |
| 89 | IFITM2 |  |  | 483 | -0.388 | -0.3230 | Yes |
| 90 | VCL |  |  | 484 | -0.388 | -0.3154 | Yes |
| 91 | RNF19A |  |  | 485 | -0.388 | -0.3078 | Yes |
| 92 | ATXN1 |  |  | 486 | -0.392 | -0.3001 | Yes |
| 93 | AP1S2 |  |  | 489 | -0.395 | -0.2967 | Yes |
| 94 | TSC22D1 |  |  | 492 | -0.400 | -0.2932 | Yes |
| 95 | ENDOD1 |  |  | 493 | -0.405 | -0.2852 | Yes |
| 96 | LDHA |  |  | 498 | -0.412 | -0.2858 | Yes |
| 97 | JUN |  |  | 503 | -0.425 | -0.2861 | Yes |
| 98 | PRSS23 |  |  | 510 | -0.438 | -0.2904 | Yes |
| 99 | CD9 |  |  | 515 | -0.443 | -0.2904 | Yes |
| 100 | CA2 |  |  | 518 | -0.448 | -0.2859 | Yes |
| 101 | AHNAK |  |  | 519 | -0.449 | -0.2771 | Yes |
| 102 | CST3 |  |  | 520 | -0.452 | -0.2682 | Yes |
| 103 | CEBPD |  |  | 523 | -0.455 | -0.2636 | Yes |
| 104 | TNFRSF21 |  |  | 526 | -0.461 | -0.2589 | Yes |
| 105 | IFITM3 |  |  | 533 | -0.477 | -0.2625 | Yes |
| 106 | ITGA6 |  |  | 535 | -0.479 | -0.2552 | Yes |
| 107 | IGFBP3 |  |  | 538 | -0.493 | -0.2499 | Yes |
| 108 | VMP1 |  |  | 542 | -0.502 | -0.2465 | Yes |
| 109 | RCAN1 |  |  | 544 | -0.505 | -0.2388 | Yes |
| 110 | COL4A1 |  |  | 545 | -0.507 | -0.2288 | Yes |
| 111 | HOPX |  |  | 546 | -0.513 | -0.2188 | Yes |
| 112 | SNHG3 |  |  | 549 | -0.518 | -0.2129 | Yes |
| 113 | GJA1 |  |  | 552 | -0.527 | -0.2069 | Yes |
| 114 | TIMP3 |  |  | 553 | -0.530 | -0.1965 | Yes |
| 115 | PLAT |  |  | 556 | -0.538 | -0.1903 | Yes |
| 116 | SPARCL1 |  |  | 557 | -0.544 | -0.1796 | Yes |
| 117 | PODXL |  |  | 559 | -0.546 | -0.1711 | Yes |
| 118 | CREM |  |  | 562 | -0.563 | -0.1644 | Yes |
| 119 | ANXA2 |  |  | 563 | -0.567 | -0.1532 | Yes |
| 120 | MT2A |  |  | 566 | -0.575 | -0.1463 | Yes |
| 121 | SPARC |  |  | 567 | -0.575 | -0.1350 | Yes |
| 122 | S100A16 |  |  | 569 | -0.582 | -0.1258 | Yes |
| 123 | LAMA4 |  |  | 571 | -0.620 | -0.1158 | Yes |
| 124 | ITPR2 |  |  | 572 | -0.634 | -0.1033 | Yes |
| 125 | VIM |  |  | 578 | -0.674 | -0.1009 | Yes |
| 126 | NEAT1 |  |  | 579 | -0.683 | -0.0875 | Yes |
| 127 | TM4SF1 |  |  | 580 | -0.689 | -0.0740 | Yes |
| 128 | S100A10 |  |  | 582 | -0.691 | -0.0626 | Yes |
| 129 | CYR61 |  |  | 584 | -0.714 | -0.0508 | Yes |
| 130 | CTGF |  |  | 588 | -0.782 | -0.0419 | Yes |
| 131 | EMP1 |  |  | 589 | -0.785 | -0.0265 | Yes |
| 132 | PMP22 |  |  | 590 | -0.837 | -0.0101 | Yes |
| 133 | CCL2 |  |  | 595 | -1.066 | 0.0022 | Yes |
Table: GSEA details [plain text format]

  

Fig 2: RICHARDS\_NOWAKOWSKI\_SCIENCE\_2017\_ENDOTHELIAL\_UPREG: Random ES distribution      
 Gene set null distribution of ES for **RICHARDS\_NOWAKOWSKI\_SCIENCE\_2017\_ENDOTHELIAL\_UPREG**
